# Editorial: Chronic inflammation, oxidative stress and lipoprotein metabolism in cardio-pulmonary continuum

**DOI:** 10.3389/fcvm.2022.1055370

**Published:** 2022-12-23

**Authors:** Alexander V. Sorokin, Hildur Arnardottir, Kazuhiko Kotani, Günther Silbernagel

**Affiliations:** ^1^Section of Inflammation and Cardiometabolic Diseases, Cardiovascular and Pulmonary Branch, National Heart, Lung and Blood Institute, National Institutes of Health, Bethesda, MD, United States; ^2^Division of Cardiovascular Medicine, Department of Medicine Solna, Center for Molecular Medicine, Karolinska Institute, Solna, Sweden; ^3^Division of Community and Family Medicine, Jichi Medical University, Tochigi, Japan; ^4^Division of Angiology, Department of Internal Medicine, Medical University of Graz, Graz, Austria

**Keywords:** inflammation, lipids, atherosclerosis, oxidation, respiratory, cardiovascular disease

Aging-associated conditions, such as cardiovascular (CVD) and respiratory diseases, represent significant public health concern (1, 2). Impaired resolution of inflammation and lipoprotein metabolism are well known risk factors of atherosclerosis development, and they often coincide with oxidative stress and respiratory dysfunction prevalent in pulmonary diseases (3). The fact that these pathologies are known to progress with age, as well as inter-connected and associate with each other, should raise further concern among cardiologists and other health care professionals. Indeed, chronic obstructive pulmonary disease (COPD), a common respiratory condition, represents the third leading cause of death worldwide (4) and epidemiological studies have shown a high prevalence of CVD comorbidities in COPD patients (5). The high rate of such association between CVD and respiratory diseases can be explained by the fact that both pathologies share common risk factors, however, underlying pathophysiological mechanisms and clinical management are still ambiguous and need further exploration.

Chronic inflammatory conditions are considered important mediators of residual cardiovascular risk (6). Indeed, in the electronic health record-based study conducted by Sinha et al., primary analyses from 17,049 subjects revealed a significantly higher risk of incident coronary heart disease in patients with systemic lupus erythematosus (SLE) (HR 2.0, 95% CI 1.2, 3.2) and systemic sclerosis (SSc) (HR 2.1, 95% CI 1.2, 3.9) as compared to subjects without chronic inflammatory conditions. Surprisingly, patients with HIV, psoriasis (PSO), rheumatoid arthritis (RA) and inflammatory bowel disease did not show the same increased risk. The observed discrepancies with previously published studies (7–10) might be related to a limited single medical system design, inclusion of individuals with mild disease and lack of specific biologic treatment records, such as in PSO patients. Though, Sinha et al. highlighted marked ethnical differences between the investigated chronic inflammatory conditions with predominant black population affected by the SLE and white population by PSO. Moreover, as expected, PSO population tended to have higher body mass index and associated diabetes as compared to other diseases. Interestingly, despite 62% of the SLE patients being on steroid therapy, incident of myocardial infarction was 4-fold higher as compared to the non-inflamed patients even after multivariate adjustment.

Moreover, Underberg et al. from the same research group, identified isolated right-sided heart failure to be more prevalent in SSc patients, whereas left ventricular systolic dysfunction was more common in HIV and SLE populations. Of notice, PSO patients did not show any significant associations with the phenotypic presentations of heart failure, which might be also attributed to the study limited data retrieval design and criteria of “possible heart failure”, which need further confirmation in larger studies.

As mentioned above, besides recognized dermatological conditions with evident immune-inflammatory component, metanalyses and national registries revealed a common presence of ischemic heart disease, heart failure and arrhythmias in subjects with respiratory conditions, such as COPD (11–13). Moreover, accompanying pulmonary hypertension and obstructive sleep apnea represent a significant health care concern (14). Although, mortality rate from COPD and cardiometabolic conditions in the US tended to decrease in male subjects over the last decades due to efficient public health policies, female population remained underrepresented in both epidemiological and clinical reports with increasing prevalence of COPD and cardiometabolic complications in the elderly population^1^.

In the review paper by Hernandez et al., the authors have expanded this important gender differences by introducing the concept of gender dimension and linking the renal system to the discussed cardio-pulmonary continuum. The authors examining importance of gender-based analyses in reporting scientific results and understanding clinical outcomes which dependent not solely on biological gender differences but also include socio-cultural characteristics. Indeed, the reported cardio-pulmonary-renal interaction is especially important during acute phase inflammatory reactions and well recognized in patients with acute respiratory distress syndrome (ARDS). In the era of SARS-CoV-2 pandemic exploring multisystemic biologic interactions dependent on gender is critical for effective treatment and disease prognosis.

By further investigating the above mentioned gender differences, Rastogi et al. elaborated on maternal obesity, which is linked to long-term morbidities, such as cardiac and pulmonary conditions, in the offspring. Besides known immune and metabolic effects associated with obesity, the authors highlighted a role of oxidative stress and microbiome disbalance during pregnancy and what future preventive and treatment measures should be applied to both mothers and children in order to prevent the cardio-pulmonary continuum from development throughout the life course.

As we tried to emphasize with the current collection of research articles, one of the reasons underlying the reported pathophysiological association between cardiovascular and pulmonary diseases resides in persistent inflammation and impaired immune response. Therefore, this impaired biological multisystem interaction also responsible for the lipoprotein metabolic dysregulation observed in chronic inflammatory conditions like PSO (15) and COPD (16). Although pharmacological reduction of LDL-C is the main tool in the primary prevention for atherosclerotic cardiovascular disease (ASCVD), other lipid targets have been recently investigated to address the issue of residual atherosclerotic risk remaining in patients with low LDL-C and high HDL-C (17). Some of these targets are modified lipoproteins produced by excessive oxidation of LDL and HDL, resulting in the formation of its oxidized forms, such as oxLDL and oxHDL, respectively. Indeed, previous studies in inflammatory diseases identified excessive amount of these lipoproteins, which were effectively decreased under specific anti-inflammatory and biologic treatment (18). The exact mechanisms and future directions of pharmaceutical inhibition of oxidized lipoproteins and related system are extensively described in the review paper by Lorey et al.

Finally, new insights into atherosclerosis biology and plaque formation have been elegantly demonstrated by Filip et al. in a model of ApoE^−/−^AOC3^−/−^ double knockout mice and postmortem human coronary plaque samples. The authors discovered that absence of amine oxidase copper containing 3 (AOC3) enzyme, involved in vascular smooth muscle cell phenotype switching, had a pro-atherosclerotic effect and led to atheroma progression.

In summary, this collection of research articles aimed to raise awareness among researchers and health care specialists of the complex interplay among pulmonary and cardiovascular diseases with immune-inflammatory component.

## Author contributions

All authors listed have made a substantial, direct, and intellectual contribution to the work and approved it for publication.

## Footnotes

^1^ https://www.cdc.gov/copd/data.html
